# Establishment of Immortalized Human Erythroid Progenitor Cell Lines Able to Produce Enucleated Red Blood Cells

**DOI:** 10.1371/journal.pone.0059890

**Published:** 2013-03-22

**Authors:** Ryo Kurita, Noriko Suda, Kazuhiro Sudo, Kenichi Miharada, Takashi Hiroyama, Hiroyuki Miyoshi, Kenzaburo Tani, Yukio Nakamura

**Affiliations:** 1 Cell Engineering Division, RIKEN BioResource Center, Tsukuba, Ibaraki, Japan; 2 Subteam for Manipulation of Cell Fate, RIKEN BioResource Center, Tsukuba, Ibaraki, Japan; 3 Department of Molecular Genetics, Division of Molecular and Clinical Genetics, Medical Institute of Bioregulation, Kyushu University, Higashi-ku, Fukuoka, Japan; University of Sao Paulo - USP, Brazil

## Abstract

Transfusion of red blood cells (RBCs) is a standard and indispensable therapy in current clinical practice. In vitro production of RBCs offers a potential means to overcome a shortage of transfusable RBCs in some clinical situations and also to provide a source of cells free from possible infection or contamination by microorganisms. Thus, in vitro production of RBCs may become a standard procedure in the future. We previously reported the successful establishment of immortalized mouse erythroid progenitor cell lines that were able to produce mature RBCs very efficiently. Here, we have developed a reliable protocol for establishing immortalized human erythroid progenitor cell lines that are able to produce enucleated RBCs. These immortalized cell lines produce functional hemoglobin and express erythroid-specific markers, and these markers are upregulated following induction of differentiation in vitro. Most importantly, these immortalized cell lines all produce enucleated RBCs after induction of differentiation in vitro, although the efficiency of producing enucleated RBCs remains to be improved further. To the best of our knowledge, this is the first demonstration of the feasibility of using immortalized human erythroid progenitor cell lines as an ex vivo source for production of enucleated RBCs.

## Introduction

The transfusion of RBCs is a standard clinical therapy. Currently, the supply of RBCs for transfusion is dependent on donation of blood by large numbers of volunteers. This system has two important shortcomings, namely, shortages of volunteers and contamination of donated blood by microorganisms. One promising way around these problems might be to produce RBCs in vitro [1], [2], [3] from hematopoietic stem/progenitor cells [4], [5], embryonic stem (ES) cells [6], or induced pluripotent stem (iPS) cells [7].

Recently, we developed a new approach in the mouse for producing RBCs in vitro [8]. Using mouse ES cells, we successfully established immortalized erythroid progenitor cell lines, which we termed mouse ES cell-derived erythroid progenitor (MEDEP) cell lines, and confirmed that these cell lines could produce mature RBCs in vitro [8]. The logical next step was to create immortalized human erythroid progenitor cell lines that could provide a convenient and reliable ex vivo source for RBC production. These cell lines could also be of value for a range of basic science investigations, for example, into erythroid differentiation and enucleation. The present study shows the feasibility of establishing immortalized human erythroid progenitor cell lines and demonstrates that enucleated RBCs can be induced to differentiate in these cell lines.

## Materials and Methods

### Cell Lines

Human iPS cell lines (HiPS-RIKEN-3A and HiPS-RIKEN-4A) and the OP9 cell line were obtained from the Cell Engineering Division of RIKEN BioResource Center (Tsukuba, Ibaraki, Japan). iPS cells were maintained in an undifferentiated state in the presence of a feeder cell line, SNL76/7, as described previously [9]. The SNL76/7 feeder cell line was obtained from the European Collection of Cell Cultures (Salisbury, Wiltshire, UK) and cultured in DMEM (Sigma, St. Louis, MO, USA) supplemented with 7.5% fetal bovine serum (FBS; Invitrogen, Carlsbad, CA, USA).

### Establishment of Human iPS Cell Lines Expressing TAL1

The internal ribosomal entry site (IRES)-puromycin resistant gene (Puro^r^) cassette was amplified by polymerase chain reaction (PCR) using pIRESpuro3 plasmid DNA (TAKARA BIO, Otsu, Shiga, Japan) with the following primers: 5′-tga tcc tct aga ctg gaa tta att cgc tgt ctg cga-3′ (sense) and 5′-gtg ggg gtt aac tca ggc acc ggg ctt gcg ggt ca-3′ (anti-sense). After confirmation of the DNA sequence, the IRES-Puro^r^ cassette was cloned into the CSII-EF-RfA lentiviral vector plasmid (Figure S1), which contains the human EF-1α promoter and the Gateway system (Invitrogen), to produce CSII-EF-RfA-IRES-Puro^r^. TAL1 cDNA and the recombination sequences for the Gateway system (Invitrogen) were amplified by reverse transcription-PCR (RT-PCR) using human fetal liver total RNA purchased from TAKARA BIO with the following primers: 5′-ggg gac aag ttt gta caa aaa agc agg ctt cac cat gac cga gcg gcc gcc gag cga-3′ (sense) and 5′-ggg gac cac ttt gta caa gaa agc tgg gtc tca ccg agg gcc ggc tcc atc ggc-3′ (anti-sense). The RT-PCR amplification product (1060 bp) was subcloned into the pDONR222 vector (Invitrogen) and verified by DNA sequencing. The TAL1 cDNA was then transferred to the CSII-EF-RfA-IRES-Puro^r^ plasmid using Gateway LR clonase (Invitrogen) to produce CSII-EF-TAL1-IRES-Puro^r^. The vesicular stomatitis virus G glycoprotein (VSV-G)-pseudotyped lentiviral vector preparation was performed as described previously [10] with the exception that Fugene HD (Roche, Mannheim, Germany) was used for transfection instead of polyethyleneimine. Human iPS cell lines were transduced with CSII-EF-TAL1-IRES-Puro^r^ lentiviral vector in the presence of polybrene (8 µg/ml; Sigma) and iPS cells expressing TAL1 were selected using puromycin (2 µg/ml; InvivoGen, San Diego, CA, USA).

### Construction of Lentiviral Vector Plasmid for Expression of Human Papilloma Virus Type 16-E6/E7 Using the Tetracycline-inducible System

The CSIV-TRE-RfA-UbC-KT lentiviral vector plasmid (Figure S2) contains the humanized Kusabira-Orange 1 (hKO1) fluorescent protein gene [11] and the reverse tetracycline (Tet)-controlled transcriptional transactivator (TAKARA BIO) linked by the Thosea asigna virus 2A peptide sequence under the control of human ubiquitin C promoter; this plasmid is designed to express cDNA under the control of the Tet-responsive promoter (TAKARA BIO). The human papilloma virus 16 (HPV16)-E6/E7 genomic DNA subcloned into pENTR201 (Invitrogen) was a kind gift of Dr. Kiyono (Division of Virology, Molecular Oncology Group, National Cancer Center Research Institute, Japan). The HPV16-E6/E7 genomic DNA was transferred to the CSIV-TRE-RfA-UbC-KT plasmid using Gateway LR clonase to produce CSIV-TRE-HPV16-E6/E7-UbC-KT. Lentiviral vector preparation was performed as described above.

### Specific Factors

The following factors were used in this study: human stem cell factor (SCF; R&D systems, Minneapolis, MN, USA), human erythropoietin (EPO; Kirin Brewery, Tokyo, Japan), human FLT3 ligand (FLT3-L; R&D systems), dexamethasone (DEX; Sigma), human vascular endothelial growth factor (VEGF; R&D systems), human insulin-like growth factor-II (IGF-II; R&D systems), and human thrombopoietin (TPO; R&D systems).

### Establishment of Immortalized Erythroid Progenitor Cell Lines from Human iPS Cells Expressing TAL1

**Figure 1:**
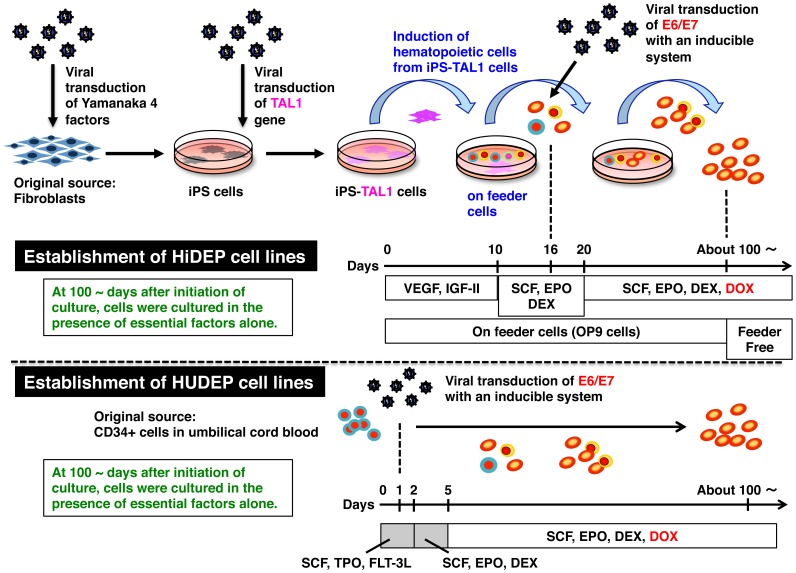
Schematic outline of the procedures for establishing immortalized human erythroid progenitor cell lines from iPS cells and from hematopoietic stem/progenitor cells in umbilical cord blood.

The protocol schedule is summarized in Table 1 and a graphical depiction of the process is shown in Figure 1. To induce hematopoietic cells from HiPS-RIKEN-3A-TAL1 and HiPS-RIKEN-4A-TAL1 cells, cells were cultured on OP9 feeder cells in basal differentiation medium composed of IMDM (Sigma) supplemented with 15% fetal bovine serum (FBS; Invitrogen), ITS (10 µg/ml human insulin, 5.5 µg/ml human transferrin, and 5 ng/ml sodium selenite; Sigma), 50 mg/mL ascorbic acid (Sigma), 0.45 mM α-monothioglycerol (Sigma), and PSQ (100 units/ml penicillin, 100 mg/ml streptomycin, and 2 mM L-glutamine; Invitrogen) in the presence of VEGF (20 ng/ml) and IGF-II (200 ng/ml). From day 10, the cells were cultured on OP9 cells in the presence of SCF (50 ng/ml), EPO (3 IU/ml) and DEX (10^−6^ M). On day 16, the HPV16-E6/E7 expression system was introduced into the cells by lentiviral transduction. Four days later, the cells were cultured on OP9 cells in the presence of DOX (1 µg/ml), SCF (50 ng/ml), EPO (3 IU/ml) and DEX (10^−6^ M). The medium was usually changed twice per week, except when cell numbers were low. Around three months after initiation of culture, the cells were able to proliferate without feeder cells. After this time point, we maintained the cells in a serum-free medium, StemSpan SFEM® medium (StemCell Technologies, Vancouver, BC, Canada), in the presence of specific factors.

**Table 1:** An outline of the culture schedule used to establish immortalized cell lines from iPS cells.

Day of culture	Feeder cells	Attached cells	Detached cells	Specific factors used	Doxycycline
Day 0	OP9	(Start)	(Start)	VEGF, IGF-II	(−)
Day 4, 7	OP9^a^	Re-cultured^b^	Discarded	VEGF, IGF-II	(−)
Day 10, 13	OP9^a^	Re-cultured^b^	Re-cultured^c^	SCF, EPO, DEX	(−)
Day 16	OP9^d^	Discarded^d^	Virus infection^e^	SCF, EPO, DEX	(−)
Day 17	OP9^a^	Re-cultured^b^	Re-cultured^c^	SCF, EPO, DEX	(−)
Day 20∼^f^	OP9a or d	Re-cultured^b^ or Discarded	Re-culturedc, g	SCF, EPO, DEX	(+)
Day 48, 97∼^f^	(−)^h^	Discarded^i^	Re-cultured^g^	SCF, EPO, DEX	(+)
Day 131∼^f^	(−)	Discarded^i^	Re-cultured^g^	SCF, EPO, DEX^j^	(+)

Human iPS cells (4x10^4^) were cultured in the presence of feeder cells with cytokines in a 100 mm dish. Attached cells indicate cells attached to the dish or to the feeder cells. Detached cells indicate cells detached from the dish or from the feeder cells. VEGF, vascular endothelial growth factor. IGF-II, insulin-like growth factor-II. SCF, stem cell factor. EPO, erythropoietin. DEX, dexamethasone.

a OP9 feeder cells were used again for a further period.

b Attached cells were cultured again on OP9 feeder cells.

c All detached cells collected from the dish were cultured again in fresh medium.

d Used OP9 cells were discarded together with any attached cells and a fresh batch of OP9 cells were used for the next culture step.

e All detached cells collected from the dish were infected with lentivirus expressing HPV16-E6/E7, and were then cultured again.

f The medium was changed twice per week.

g Detached cells from the dish were either all re-cultured or some re-cultured and the remainder stored for further analyses.

h From this time point, the detached cells could proliferate without feeder cells.

i Very few attached cells were detected.

j At approximately day 131, the requirement for essential factors for proliferation was evaluated.

### Establishment of Immortalized Erythroid Progenitor Cell Lines from Human CD34-positive Hematopoietic Stem/progenitor Cells

The protocol schedule is summarized in Table 2 and a graphical depiction of the process is shown in Figure 1. CD34-positive hematopoietic stem/progenitor cells derived from umbilical cord blood were obtained from the Stem Cell Resource Network in Japan (Banks at Miyagi, Tokyo, Kanagawa, Aichi, and Hyogo) through the RIKEN BioResource Center. CD34-positive cells (1×10^5^ cells) were cultured in a serum-free medium, StemSpan SFEM® medium (StemCell Technologies), in the presence of SCF (50 ng/ml), TPO (50 ng/ml) and FLT3-L (50 ng/ml). On day 1 of culture, the HPV16-E6/E7 expression system was introduced into the cells by lentiviral transduction. Four days after introduction of the HPV16-E6/E7 expression system, the cells were cultured in the presence of doxycycline (DOX, 1 µg/ml; TAKARA BIO), SCF (50 ng/ml), EPO (3 IU/ml) and DEX (10^−6^ M). DOX was used as a substitute for Tet, and the presence of DOX induced the expression of HPV16-E6/E7. The medium was usually changed twice per week, except when cell numbers were low.

**Table 2:** An outline of the culture schedule used to establish immortalized cell lines from hematopoietic stem/progenitor cells.

Day of culture	Attached cells	Detached cells	Specific factors used	Doxycycline
Day 0	(Start)	(Start)	SCF, TPO, FLT3-L	(−)
Day 1	Not detected	Virus infection^a^	SCF, TPO, FLT3-L	(−)
Day 2	Not detected	Re-cultured^b^	SCF, EPO, DEX	(−)
Day 5, 8	Discarded^c^	Re-cultured^b^	SCF, EPO, DEX	(+)
Day 11∼^d^	Discarded^c^	Re-cultured^e^	SCF, EPO, DEX	(+)
Day 110∼^d^	Discarded^c^	Re-cultured	SCF, EPO, DEX^f^	(+)

CD34^+^ cells (1×10^5^) were cultured in the absence of feeder cells with cytokines in the well of a 24-well plate or in a 100 mm dish. SCF, stem cell factor. EPO, erythropoietin. FLT3-L, FLT3 ligand. DEX, dexamethasone.

a All cells collected from the dish were infected with lentivirus expressing HPV16-E6/E7 and then cultured again.

b All cells collected from the dish were cultured again.

c Very few attached cells were detected.

d The medium was changed twice per week.

e Detached cells collected from the dish were either all cultured again or only some were re-cultured and the remainder were stored for further analysis.

f At approximately Day 110, the cultures were evaluated for the essential factor(s) for proliferation.

### Induction of Differentiation of Immortalized Erythroid Progenitor Cell Lines

The immortalized erythroid progenitor cell lines were induced to differentiate into more mature erythroid cells by culture in erythroid differentiation medium; IMDM (Sigma) containing 10% human AB serum (Kohjin Bio, Saitama, Japan or TAKARA BIO), α-tocopherol (20 ng/ml; Sigma), linoleic acid (4 ng/ml; Sigma), cholesterol (200 ng/ml; Sigma), sodium selenite (2 ng/ml; Sigma), holo-transferrin (200 µg/ml; Sigma), human insulin (10 µg/ml; Sigma), ethanolamine (10 µM; Sigma), 2-ME (0.1 mM; Sigma), D-mannitol (14.57 mg/ml; Sigma), mifepristone (an antagonist of glucocorticoid receptor, 1 µM; Sigma) and EPO (5 IU/ml).

### Flow Cytometry

A sample of cells (<1×10^6^) was stained with monoclonal antibodies (MoAbs) in 100 µl of staining medium (phosphate buffered saline [PBS; Sigma] containing 2% FBS [Invitrogen] and 0.25% sodium azide [Sigma]) for 30 min on ice. The cells were washed twice with the staining medium, and analyzed using a FACS Calibur (BD Biosciences, San Jose, CA, USA). MoAbs against human antigens were conjugated with fluorescein isothiocyanate (FITC), Alexa Fluor® 488 (Alexa488) or allophycocyanin (APC). FITC-conjugated CD34 (cat. #555821), CD36 (cat. #555454), CD41a (cat. #555466), and CD71 (cat. #555536), Alexa488-conjugated CD11b (cat. #557701) and APC-conjugated CD33 (cat. #551378), CD45 (cat. #555485), c-KIT (cat. #550412), glycophorin A (GPA) (cat. #551336) and HLA-ABC (cat. #555555) were purchased from BD Biosciences. FITC-conjugated mouse IgG_1_ (cat. #555748), IgM (cat. #555583), APC-conjugated mouse IgG_1_ (cat. #555751) and IgG_2b_ (cat. #555745) were also purchased from BD Biosciences and used as isotype controls. Cell viability was monitored by propidium iodide (PI; Sigma) staining. Flow cytometry data was analyzed using CellQuest analysis software (BD Biosciences). In this analysis, PI-positive cells were excluded as being dead and PI-negative cells were analyzed as being viable.

### Functional Analysis of Hemoglobin

Following induction of differentiation of immortalized erythroid progenitor cells (5×10^7^ cells) for 4 to 6 days, the cells were collected, washed with PBS, and subjected to analysis. Oxygen equilibrium curves were determined using a Hemox-Analyzer Model B (TSC Scientific, New Hope, PA). The gas phase gradients were obtained using nitrogen and room air, and the curves were run in both directions. Human peripheral blood cells and umbilical cord blood cells were used as the controls.

### Quantitative Reverse Transcription-polymerase Chain Reaction (qRT-PCR)

Total RNA was extracted from cells using ISOGEN™ reagent (Wako, Osaka, Japan). Reverse transcription was carried out using 4.5 µg total RNA and the SuperScript®III First Strand Synthesis System (Invitrogen) in a 20 µl reaction volume. After reverse transcription, 180 µl of water was added to the reaction mixture and a 1 µl aliquot was used for each PCR. PCR was carried out using FastStart TaqMan® Probe Master and Universal ProbeLibrary Probes (Roche, Mannheim, Germany). PCR products were monitored by FAM (CarboxyFluorescein) dye fluorescence using a Thermal Cycler Dice® Real Time System (Takara Bio). Glyceraldehyde-3-phosphate dehydrogenase (GAPDH) amplification was used as the internal control.

### Primers and Probes

The following primers and probes were used in this study: human GATA1, sense primer 5′-cac tga gct tgc cac atc c-3′ and antisense primer 5′-atg gag cct ctg ggg att a-3′ (Probe #26); human GATA2, sense primer 5′-aag gct cgt tcc tgt tca ga-3′ and antisense primer 5′-ggc att gca cag gta gtg g-3′ (Probe #70); human GFI1B, sense primer 5′-cct ctt gtg ccc agc act-3′ and antisense primer 5′-cgt gag ggg tgg aga aga c-3′ (Probe #41); human EKLF, sense primer 5′-aca cca aga gct ccc acc t-3′ and antisense primer 5′-gta gtg gcg ggt cag ctc-3′ (Probe #7); human EPO receptor (EPOR), sense primer 5′-ttg gag gac ttg gtg tgt ttc-3′ and antisense primer 5′-agc ttc cat ggc tca tcc t-3′ (Probe #69); human α-globin, sense primer 5′-gac ccg gtc aac ttc aag c-3′ and antisense primer 5′-aga agc cag gaa ctt gtc ca-3′ (Probe #10); human β-globin, sense primer 5′-gca cgt gga tcc tga gaa ct-3′ and antisense primer 5′-cac tgg tgg ggt gaa ttc tt-3′ (Probe #61); human γ-globin, sense primer 5′-tgg atc ctg aga act tca agc-3′ and antisense primer 5′-gcc act gca gtc acc atc t-3′ (Probe #72); human c-MYB, sense primer 5′-agc aag gtg cat gat cgt c-3′ and antisense primer 5′-gat cac acc atg atg aag aat cag-3′ (Probe #37); human SOX6, sense primer 5′-gct tct gga ctc agc cct tt-3′ and antisense primer 5′-gga gtt gat ggc atc ttt gc-3′ (Probe #67); human BCL11A, sense primer 5′-ccc aaa cag gaa cac ata gca-3′ and antisense primer 5′-gag ctc cat gtg cag aac g-3′ (Probe #52); human TAL1 (to detect endogenous TAL1 gene), sense primer 5′-tgt gtg aga gac ggt gtc ttg-3′ and antisense primer 5′-caa ggc tgc aga cag caa-3′ (Probe #19); human Band 3, sense primer 5′-tct tca gga acg tgg agc tt-3′ and antisense primer 5′-cct cat caa agg ttg cct tg-3′ (Probe #89); human Band 4.1, sense primer 5′-cca cac tga gac caa gac ca-3′ and antisense primer 5′-cca agt ctc cac tgt tgt cgt-3′ (Probe #44); human Ankyrin-1, sense primer 5′-gag cac gag gag gtg act gt-3′ and antisense primer 5′-gtg tcg agg tgt gat cct tg-3′ (Probe #65); human α-Spectrin, sense primer 5′-gct ttg aaa ggg acc tcg ta-3′ and antisense primer 5′-ctc tgc tgt ctc ccc cag t-3′ (Probe #14); human GAPDH, sense primer 5′-agc cac atc gct cag aca c-3′ and antisense primer 5′-gcc caa tac gac caa atc c-3′ (Probe #60).

### Morphological Analysis and Cell Staining

Cell morphologies were assessed using smears prepared on microscope slides or attached to microscope slides using a Cytospin 3 (Thermo Electron Corporation, Waltham, MA, USA). The cells were stained with Diff-Quik (Sysmex International, Kobe, Japan) and analyzed by microscopy.

Cell size and cell viability were measured using an automated cell counter, ViCell™ (Beckman Coulter, Fullerton, CA, USA).

Supravital staining was performed by incubating the cells in 0.3% new methylene blue solution (Muto Pure Chemicals, Tokyo, Japan) at room temperature for 20 min.

Immunostaining with GPA antibody was performed as reported previously [12]. Cells were spun onto microscope slides, washed with PBS(−) (PBS free of Ca and Mg) and fixed in 2% paraformaldehyde/PBS on ice for 10 min. After fixation, the cells were washed three times with PBS(−). Nonspecific binding was blocked with 2% BSA/PBS for 1–3 hr. Samples were incubated at 4°C overnight with mouse monoclonal biotinylated anti-human GPA antibody (R&D systems) diluted to 1∶200 in blocking solution. At the same time, SYTO16 solution (Invitrogen), which is a cell membrane-permeable fluorochrome dye that stains nucleic acids, was added to the staining mixture at a final concentration of 0.5 µM. After extensive washing with PBS(−), the cells were incubated with Alexa 647-conjugated streptavidin for 30 min to 1 hr to detect the biotinylated anti-human GPA antibody bound to the cells. After washing, the cells were observed with an LSM780 laser scanning microscope (Carl Zeiss, Oberkochen, Germany).

Benzidine staining was performed using the Peroxidase Stain DAB Kit (Nacalai Tesque, Kyoto, Japan) according to the manufacturer’s instructions. After staining, coverslips were mounted using Vectashield containing DAPI (Vector Laboratories, Burlingame, CA, USA) in order to identify nucleated and enucleated cells.

## Results

### Establishment of Immortalized Erythroid Progenitor Cell Lines from Human iPS Cells

Initially, we attempted to establish immortalized human erythroid progenitor cell lines from human ES cells and iPS cells using essentially the same protocol as for the successful establishment of MEDEP cell lines from mouse ES cells [8]. Three human ES cell lines and two human iPS cell lines were used in these experiments but none yielded abundant erythroid cells although some hematopoietic cells were induced. Thus, the protocol for establishing MEDEP cell lines failed to establish immortalized erythroid progenitor cell lines from human pluripotent stem cell lines.

**Figure 2:**
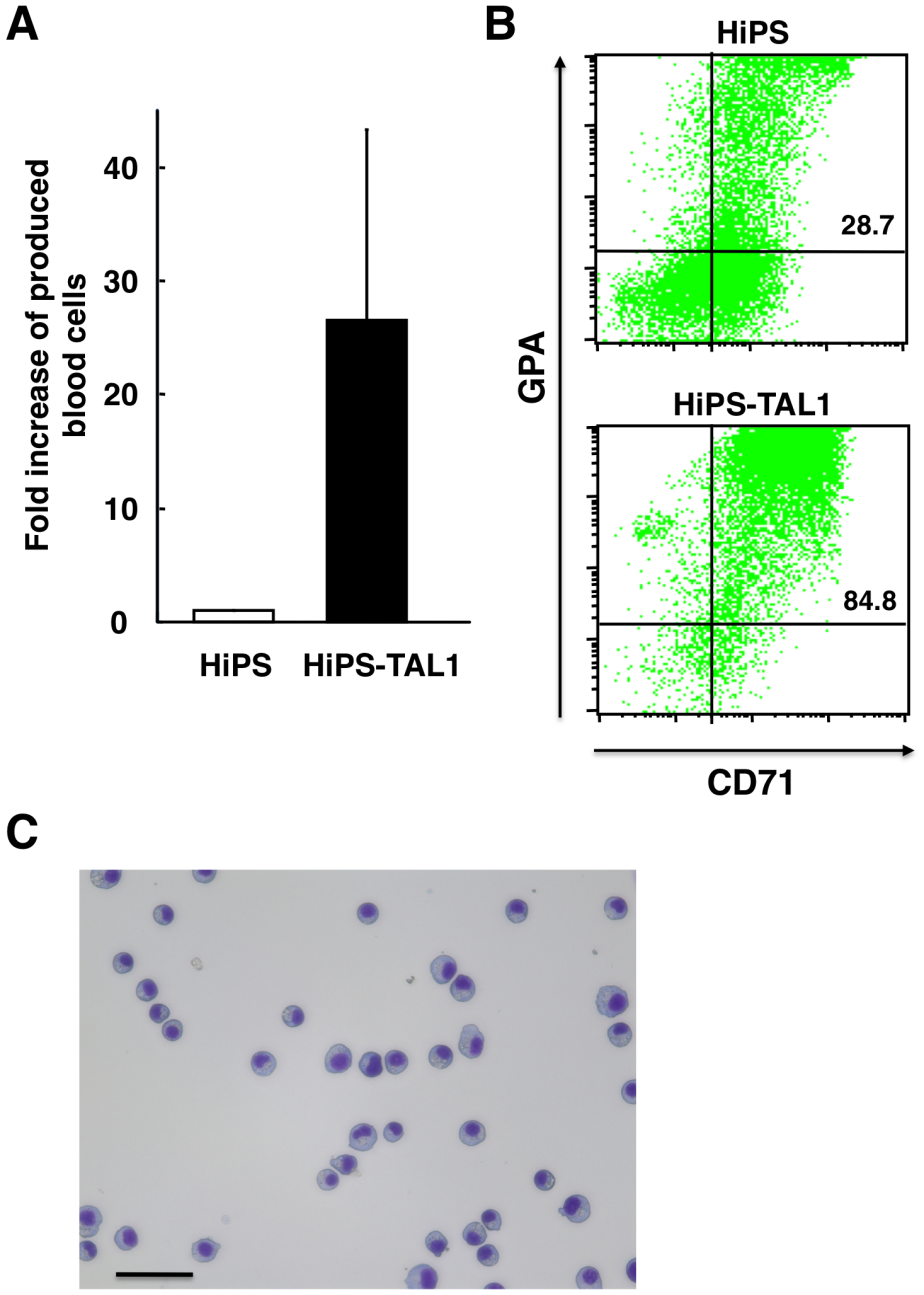
Effect of enforced expression of a transcription factor, TAL1, on induction of hematopoietic cells from human iPS cells. HiPS, human iPS cells (HiPS-RIKEN-3A). HiPS-TAL1, HiPS cells expressing TAL1 (HiPS-RIKEN-3A-TAL1). The cells were analyzed after the induction of differentiation of hematopoietic cells for 15 days. (A) Fold increase of production of hematopoietic cells from HiPS-TAL1 cells compared to HiPS cells. (B) Flow cytometer analysis. CD71, transferrin receptor. Glycophorin A (GPA), an erythroid specific marker. Percentages of GPA-positive cells are indicated in the figure. (C) Morphology of the cells derived from HiPS-TAL1 cells. Scale bar indicates 50 µm. The comparison of HiPS-RIKEN-4A and HiPS-RIKEN-4A-TAL1 showed similar results.

Next, we investigated whether enforced expression of the transcription factor TAL1 might enable establishment of immortalized erythroid progenitor cell lines. TAL1 plays essential roles in early hematopoiesis [13] and differentiation of erythroid cells and megakaryocytes [14], [15]. In an earlier study, we also showed that its expression improved the efficiency of inducing hematopoietic cells from common marmoset ES cells [12]. Therefore, we forced expression of TAL1 in human iPS cell lines (HiPS) [16] and established sub-lines, HiPS-TAL1. HiPS-TAL1 cells showed a significant improvement in the efficiency of induction of hematopoietic cells when grown on OP9 feeder cells in the presence of insulin-like growth factor-II (IGF-II) and vascular endothelial growth factor (VEGF) (Figure 2). Long-term cultures of HiPS-TAL1 cells were also initiated on OP9 cells in the presence of stem cell factor (SCF), erythropoietin (EPO) and thrombopoietin (TPO). The cells proliferated continuously and no longer required OP9 cells after they had been in culture for approximately three months. As a result, we were able to establish six immortalized cell lines that proliferated continuously for more than 1 year. However, these cell lines expressed few hematopoietic cell markers and did not differentiate into more mature cells (data not shown).

Immortalized erythroid progenitor cell lines can be established by transformation of erythroid progenitor cells with the HPV16-derived proteins HPV16-E6/E7 [17]. These cell lines do not produce mature erythrocytes, such as enucleated RBCs, possibly because continuous expression of HPV16-E6/E7 inhibits terminal differentiation of the cells.

In order to test this possibility, we used a Tet-inducible expression system [18], [19] to control expression of HPV16-E6/E7 in iPS-derived hematopoietic progenitor cells and investigated whether such expression inhibited establishment of immortalized erythroid progenitor cell lines. Hematopoietic cells were induced from HiPS-TAL1 cells that had been in culture for 16 days (see Materials and methods); the cells were infected with the lentiviral vector containing the Tet-inducible expression system for HPV16-E6/E7. One day after viral infection, the cells were transferred to a new culture on OP9 cells in the presence of SCF, EPO and dexamethasone (DEX) for 3 days. The cells were then cultured in the presence of SCF, EPO, DEX and doxycycline (DOX) over a prolonged period with regular changes of medium. DOX was used as a substitute for Tet, and the presence of DOX induced expression of HPV16-E6/E7. The cells proliferated continuously for more than 1 year; thus, we succeeded in establishing immortalized cell lines. Two cell lines were established from two independent trials using two different HiPS-TAL1 cell lines. We designated these cells as human iPS cell-derived erythroid progenitor (HiDEP) cell lines. HiDEP-1 and HiDEP-2 were established from HiPS-RIKEN-4A and HiPS-RIKEN-3A, respectively.

### Establishment of Immortalized Erythroid Progenitor Cell Lines from CD34-positive Hematopoietic Cells in Human Umbilical Cord Blood

As it would be more convenient if immortalized human erythroid progenitor cell lines could be established without recourse to ES or iPS cells, we investigated whether our protocol could be applied to the CD34-positive hematopoietic stem/progenitor cells in umbilical cord blood. CD34-positive cells were collected from umbilical cord blood and cultured in the presence of SCF, TPO and FLT3-ligand (FLT3-L) for 1 day; the cells were then infected with the lentiviral vector containing the Tet-inducible expression system for HPV16-E6/E7. One day after viral infection, the cells were cultured with SCF, EPO and DEX for 4 days. Non-adherent cells were then collected and cultured in the presence of SCF, EPO, DEX and DOX over a prolonged period with regular changes of medium. The cells proliferated continuously for more than 1 year; thus, we also succeeded in establishing immortalized hematopoietic cell lines from umbilical cord blood cells as well. In total, three cell lines were established from three independent trials using three different cord blood samples. We designated these cells as human umbilical cord blood-derived erythroid progenitor (HUDEP) cell lines, HUDEP-1, HUDEP-2 and HUDEP-3.

### Dependency on Externally Supplied Culture Factors

**Figure 3:**
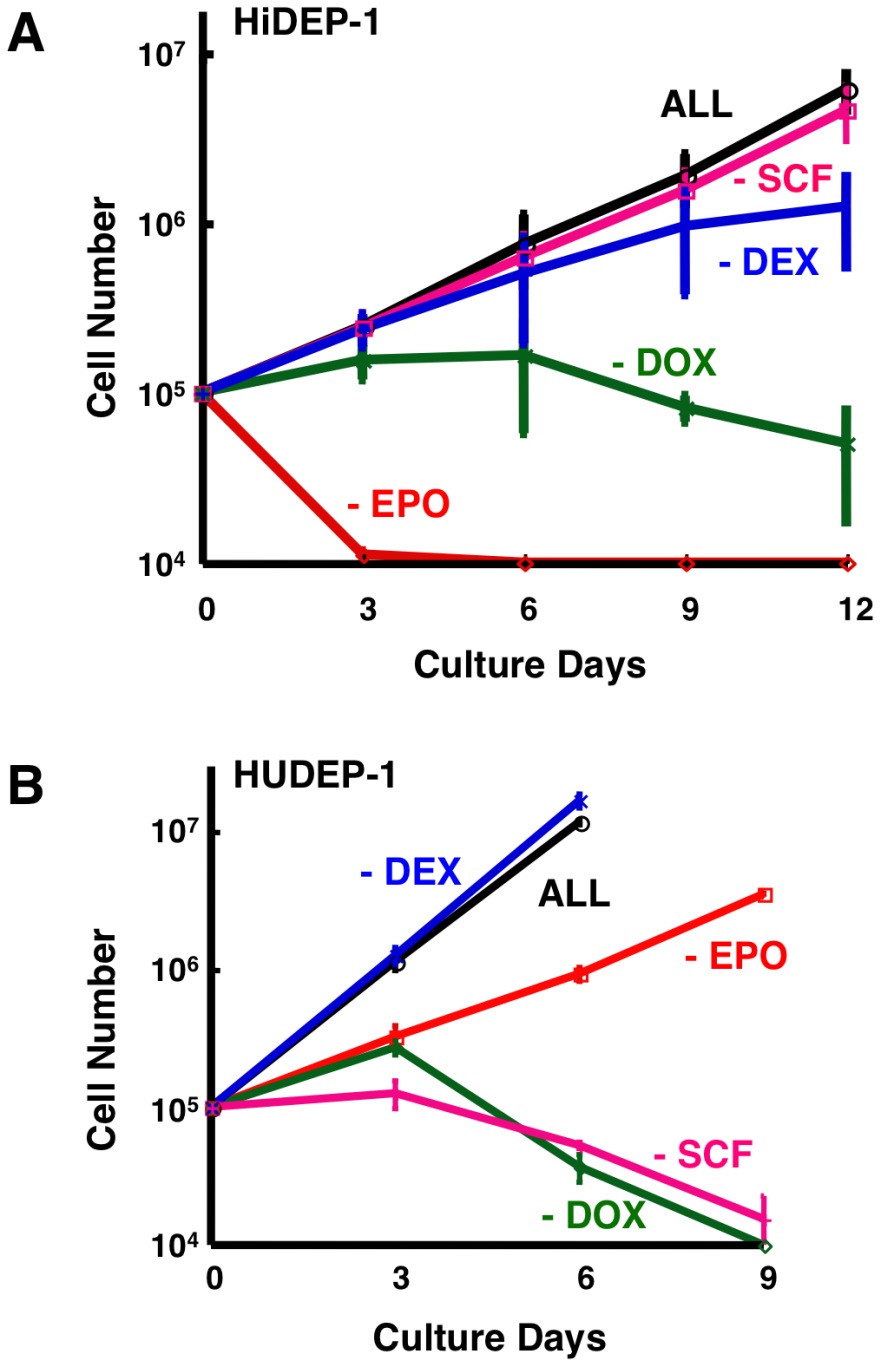
Dependency of the established erythroid progenitor cell lines on externally supplied culture factors. (A) The survival and proliferation of HiDEP-1 cells are dependent on DOX (HPV16-E6/E7) and EPO and partially dependent on DEX. (B) The survival and proliferation of HUDEP-1 cells are dependent on DOX (HPV16-E6/E7) and SCF and partially dependent on EPO. (A, B) DOX, doxycycline; expression of HPV16-E6/E7 is induced by DOX. SCF, stem cell factor. EPO, erythropoietin. DEX, dexamethasone. ALL, cells were cultured in the presence of DOX, SCF, EPO and DEX. –DOX, –SCF, –EPO, –DEX, cells were cultured after deprivation of DOX, SCF, EPO and DEX, respectively. Dependencies of other cell lines on externally supplied culture factors are summarized in Table S1.

The HiDEP and HUDEP cell lines varied in their dependency on externally supplied culture factors (Table S1); thus, for example, the survival and proliferation of HiDEP-1 cells were dependent on DOX (HPV16-E6/E7) and EPO, and partially dependent on DEX but not on SCF (Figure 3A) and the HUDEP-1 cells were dependent on DOX and SCF and partially dependent on EPO but not on DEX (Figure 3B). After confirmation of these dependencies, all cell lines were cultured in the presence of essential factors alone for each cell line.

To date, all of the cell lines have continued to proliferate vigorously with no indication of slowdown in cell division rates. We also confirmed that cultures could be re-established after freeze-thaw cycles for all cell lines. Below, we describe the characteristics of the cell lines after continuous culture for more than 6 months and after more than 80 cell division cycles.

### Expression of Cell Surface Molecules

**Figure 4:**
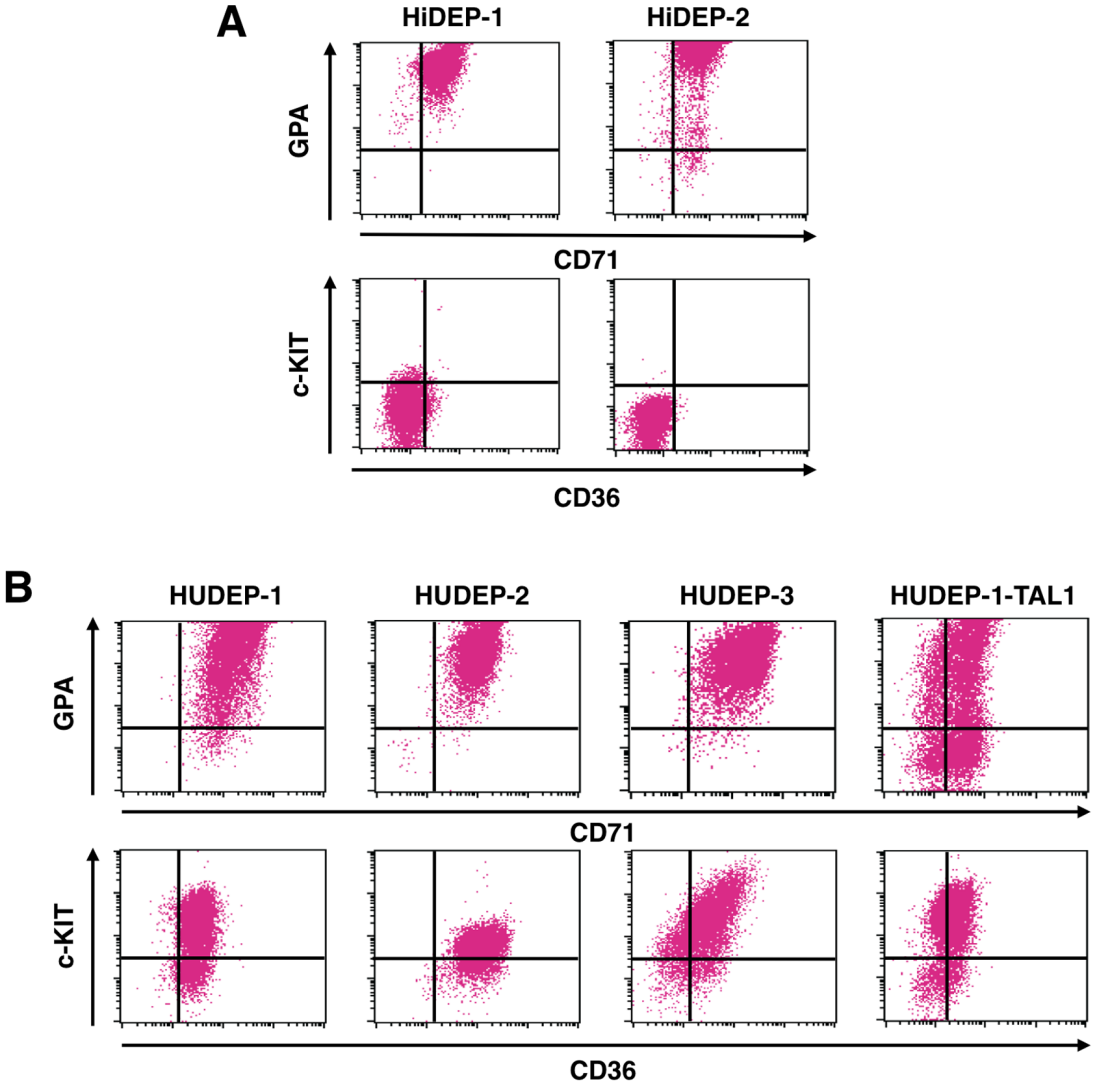
Flow cytometer analyses of the established erythroid progenitor cell lines. (A) Representative results of HiDEP cells. (B) Representative results of HUDEP cells and HUDEP-1 expressing TAL1, HUDEP-1-TAL1. (A, B) GPA, glycophorin A. CD71, transferrin receptor. c-KIT, the receptor of SCF. CD36, a marker of immature erythroid cells.

Flow cytometric analyses demonstrated that both HiDEP-1 and HiDEP-2 cells expressed the erythroid-specific cell surface marker glycophorin A (GPA) at a high level (Figure 4A), while other hematopoietic cell markers, such as CD11b, CD33, CD34, CD41a and CD45, were not detectable (Figure S3A). CD36 and c-KIT (CD117), markers of immature erythroid cells, were detected at very low levels (Figure 4A and Figure S3A).

All three HUDEP cell lines expressed CD71, glycophorin A, CD36 and c-KIT (Figure 4B). In contrast to the HiDEP cell lines, HUDEP cell lines expressed CD33 and CD45 at various levels, although non-erythroid markers such as CD11b and CD41a were not detectable (Figure S3B). This difference between HiDEP and HUDEP cells might be due to the fact that HiDEP cell lines were established from cells expressing TAL1. To determine whether this was the case, we forced expression of TAL1 in HUDEP-1 cells at 7 days after induction of HPV16-E6/E7 expression and established the cell line HUDEP-1-TAL1. However, the phenotype and characteristics of HUDEP-1-TAL1 cells were quite similar to those of HUDEP-1 cells (Figure 4B and Figure S3B).

### Induction of Differentiation

Next, we examined whether HiDEP and HUDEP cells could differentiate into more mature stages and produce enucleated RBCs. We found that differentiation could be induced by culturing the HiDEP and HUDEP cells in an erythroid differentiation medium in the presence of EPO alone (see Materials and methods).

### Production of Hemoglobin

**Figure 5:**
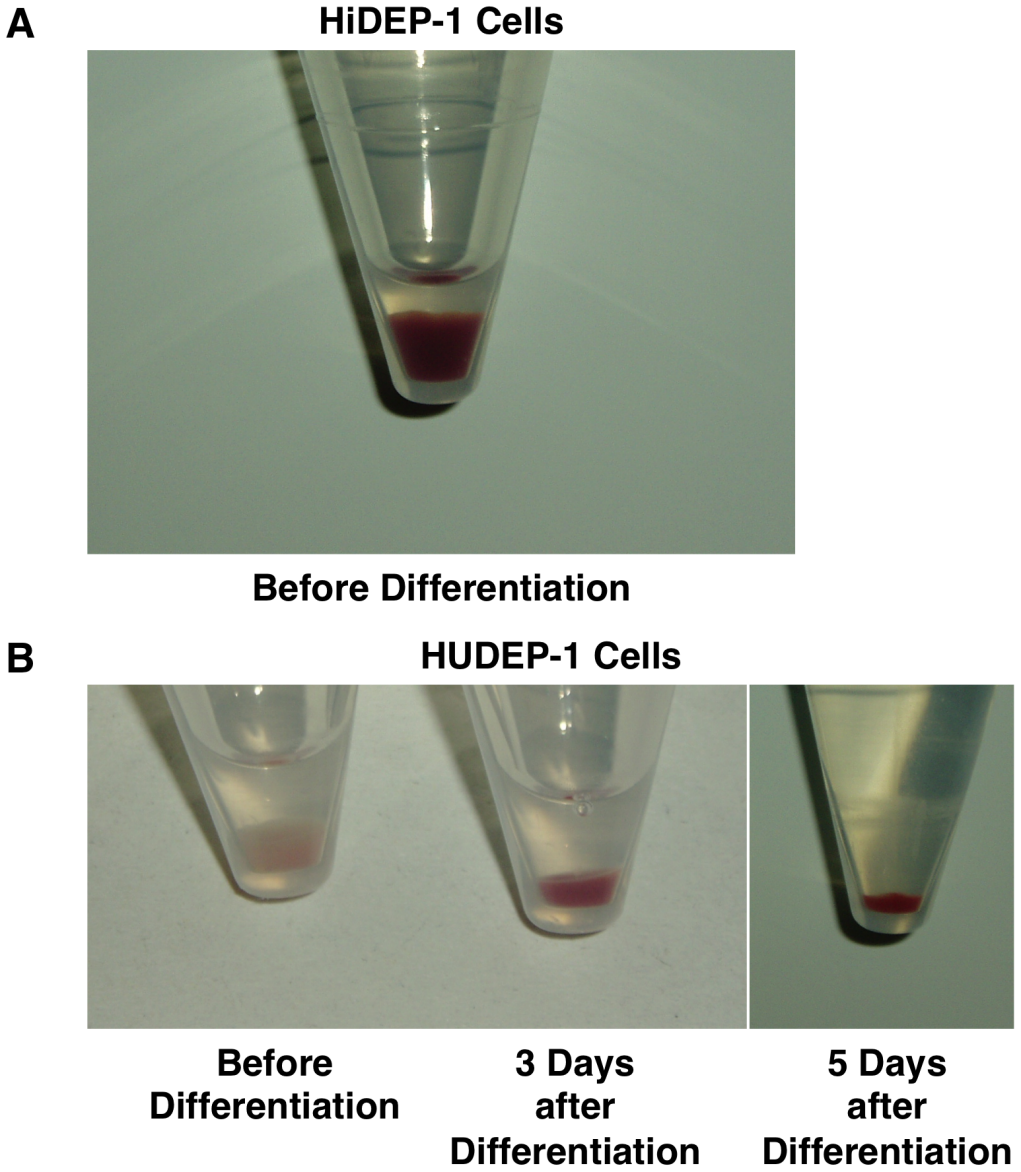
Cell pellets of the established erythroid progenitor cell lines. (A) HiDEP-1 cells before the induction of differentiation. (B) HUDEP-1 cells before and after the induction of differentiation. HUDEP-1 cells were cultured in erythroid differentiation medium on OP9 feeder cells to maintain cell viability during the differentiation process. All other cell lines also showed red cell pellets after the induction of differentiation.

Upon centrifugation, the HiDEP-1 cells produced a red cell pellet even before the induction of differentiation (Figure 5A) suggesting a continuous and abundant production of hemoglobin. In contrast, upon centrifugation, the HUDEP-1 cells yielded a light orange cell pellet before the induction of differentiation and a red cell pellet after the induction of differentiation (Figure 5B) suggesting that hemoglobin synthesis was upregulated following differentiation.

### Functional Analysis of Hemoglobin Produced in HiDEP and HUDEP Cells

**Figure 6:**
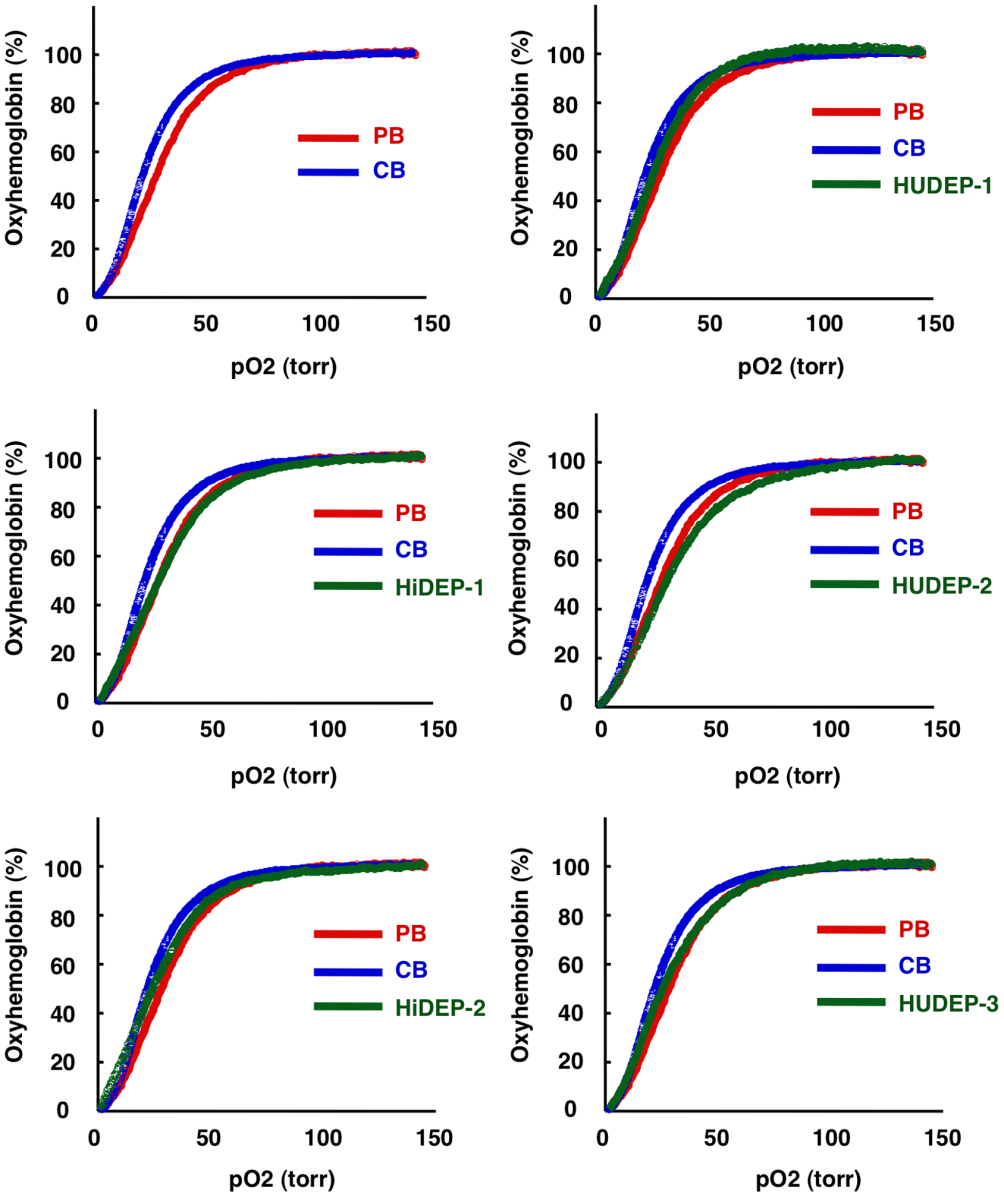
Oxygen-carrying abilities of hemoglobin produced in the established erythroid progenitor cell lines. Oxygen equilibrium curves were determined using an automated apparatus. Following the induction of differentiation, the cells were subjected to the analyses. CB, umbilical cord blood. PB, peripheral blood of adult.

We used a Hemox-Analyzer to measure the oxygen binding and dissociation abilities of the hemoglobin produced in mature cells derived from the HiDEP and HUDEP cells [20], [21]. Although the oxygen binding and dissociation curves obtained with HiDEP- and HUDEP-derived cells differed between cell lines, they showed similar curves to those obtained with adult peripheral blood or umbilical cord blood (Figure 6). The variation among the curves may be due to the types of hemoglobin expressed in each cell line, i.e., adult type hemoglobin, fetal type hemoglobin or mixture of both types (see below). Apart from this variation, the data indicate that HiDEP and HUDEP cells can produce hemoglobin with oxygen binding and dissociation abilities equivalent to red blood cells produced in vivo.

### Analysis of Gene Expression

**Figure 7:**
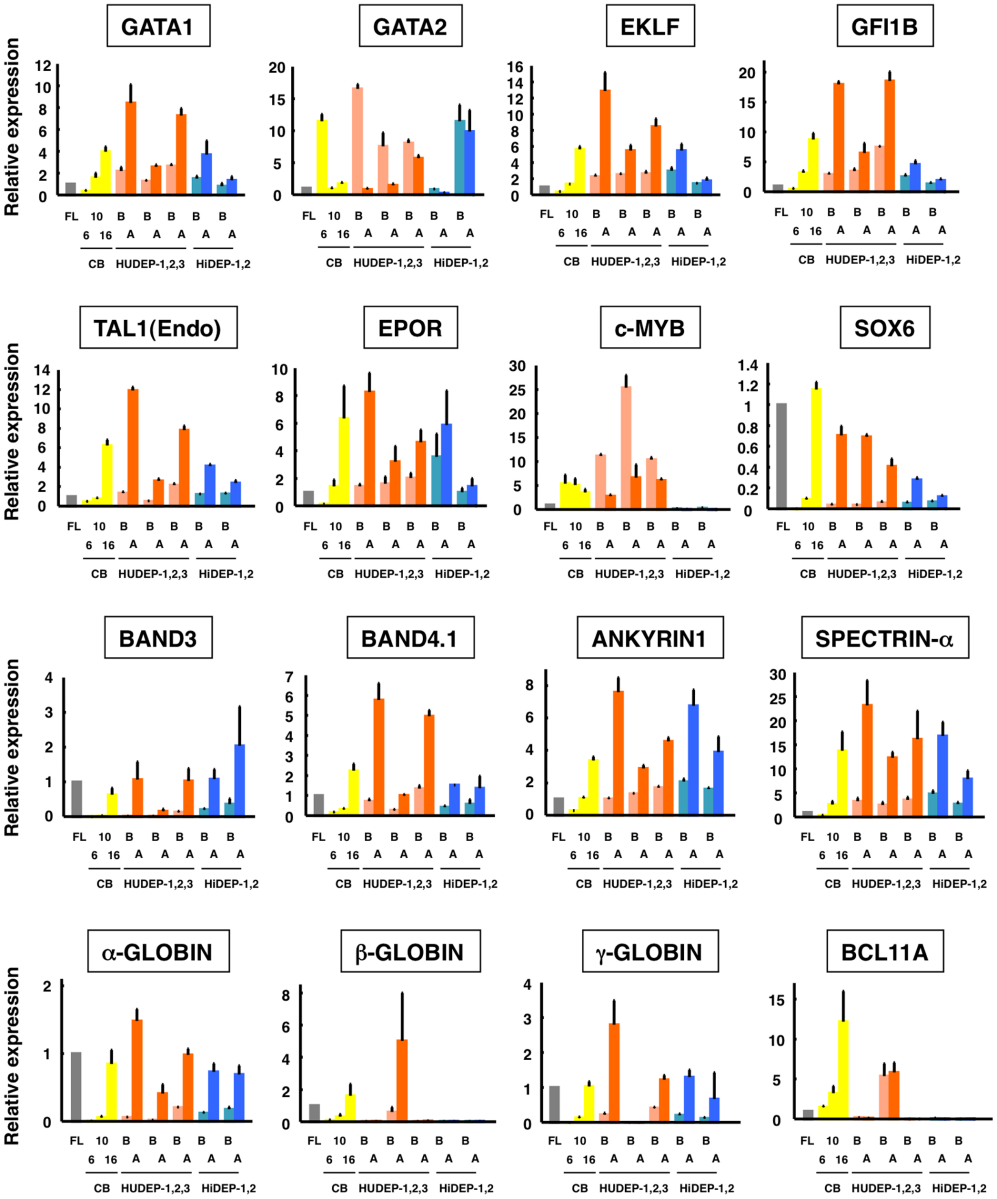
Gene expression profiles of the established erythroid progenitor cell lines estimated by quantitative RT-PCR analysis. Analyzed genes are indicated. FL, results with cDNA derived from human fetal liver. Relative expression was evaluated compared to that of FL. CB, cord blood. CB 6, 10 and 16, results with cDNA derived from cultured erythroid cells, i.e., CD34-positive cells in CB were induced to differentiate into mature erythroid cells for 6, 10 and 16 days, respectively, using the previously reported method [4]. B and A, Before and 2 days after induction of differentiation of HiDEP and HUDEP cells.

Gene expression profiles were analyzed by quantitative RT-PCR (qRT-PCR) before and after the induction of differentiation in the HiDEP and HUDEP cells lines. The erythroid-specific markers GATA1, EKLF, GFI1B, TAL1 and EPOR were detected in all cell lines and their expression profiles before and after differentiation were similar to those of cultured erythroid cells derived from umbilical cord blood (Figure 7). This was also the case for c-MYB and SOX6, which are markers of definitive erythroid cells [22], [23], [24]. In addition, we detected upregulation of the erythroid membrane genes, Band 3, Band 4.1, Ankyrin-1 and α-Spectrin in all cell lines after differentiation (Figure 7).

After differentiation, we found upregulation of α-globin in all cell lines, upregulation of β-globin only in the HUDEP-2 cell line, and upregulation of γ-globin in all cell lines except HUDEP-2. Of note, BCL11A, a repressor of γ-globin expression [25] and critical mediator of globin switching [26], was abundantly detected only in the HUDEP-2 cell line in which β-globin was up-regulated after differentiation (Figure 7).

### Cell Viability and Cell Size

**Figure 8:**
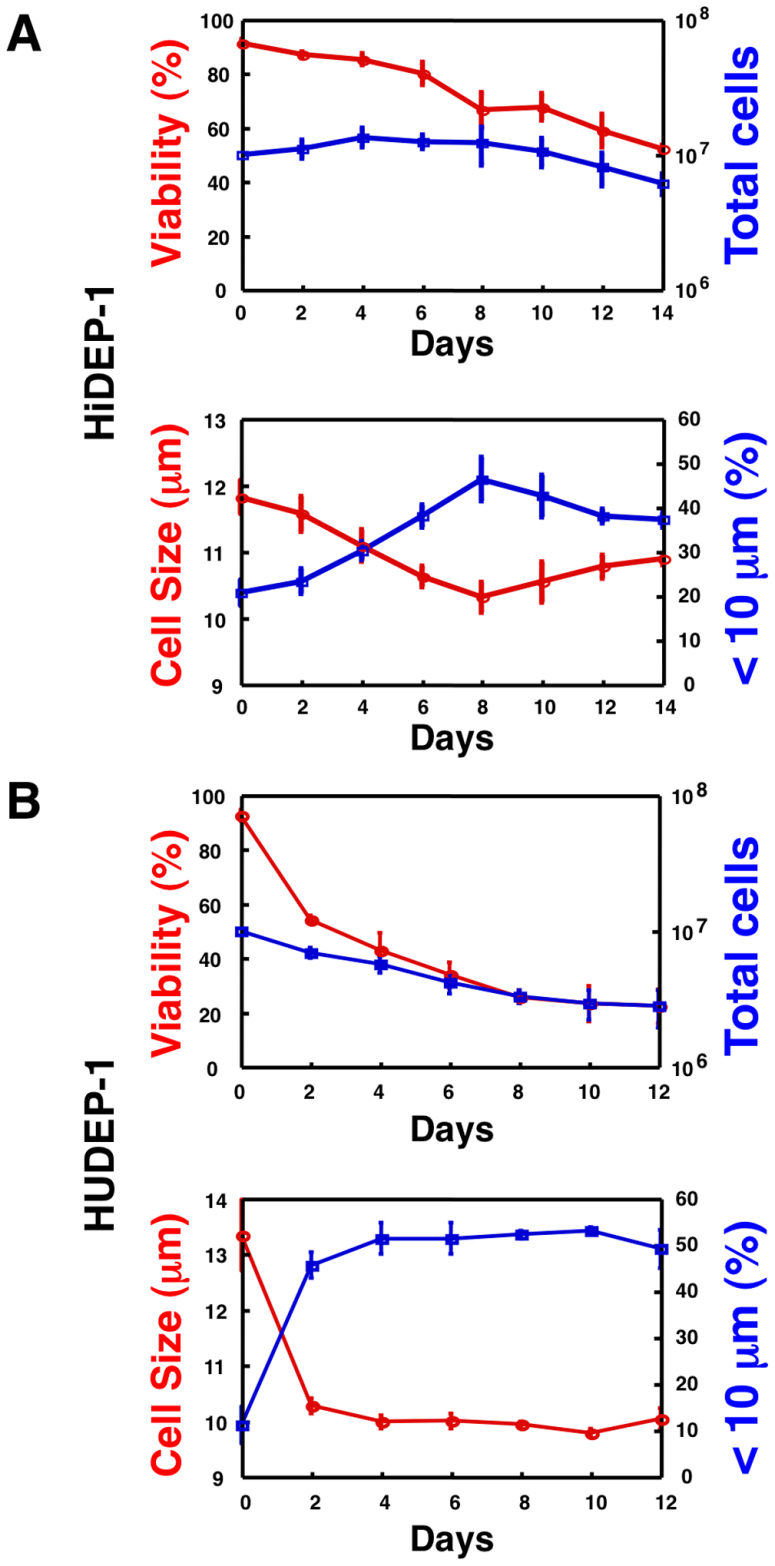
Analyses of cell viability and cell size during the induction of differentiation of the established erythroid progenitor cell lines. Cell viability and cell size were estimated by an automated cell counter. (A) Results of HiDEP-1 cells. (B) Results of HUDEP-1 cells. (A, B) <10 µm (%), the percentages of cells with a diameter less than 10 µm. Among all HiDEP and HUDEP cell lines, HiDEP-1 cells most efficiently differentiated into more mature cells.

After the induction of differentiation in HiDEP-1 cells, the proportion of viable cells decreased but more than half of the cells were still viable 14 days after differentiation (Figure 8A). The average cell size after differentiation decreased compared to undifferentiated cells and the proportion of cells with a diameter less than 10 µm increased from about 20% to approximately 40% (Figure 8A) indicating the increase of very mature erythroid cells.

After the induction of differentiation in HUDEP-1 cells, the frequency of viable cells was considerably reduced compared to HiDEP-1 cells (Figure 8B). However, among viable cells average cell sizes decreased rapidly for two days after differentiation compared to undifferentiated cells, and the proportion of cells with a diameter less than 10 µm increased from about 10% to about 50% (Figure 8B) indicating the increase of very mature erythroid cells. To improve viability, the cells were cultured on OP9 feeder cells and a morphological analysis was performed.

### Morphological Changes during Differentiation

**Figure 9:**
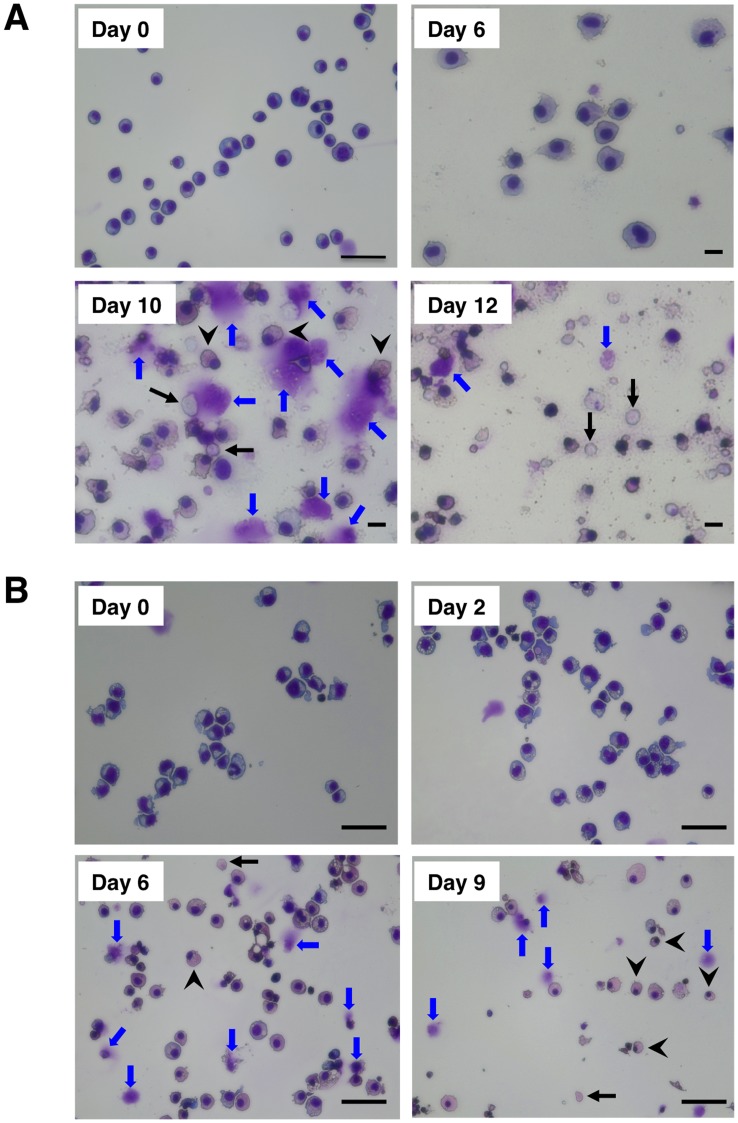
Morphological analyses during the induction of differentiation of the established erythroid progenitor cell lines. (A) HiDEP-1 cells before (Day 0) and 6, 10 and 12 days after the induction of differentiation (Day 6, 10 and 12). Scale bar in Day 0 indicates 50 µm and other scale bars indicate 10 µm. (B) HUDEP-1 cells before (Day 0) and 2, 6 and 9 days after the induction of differentiation (Day 2, 6 and 9). HUDEP-1 cells were cultured in erythroid differentiation medium on OP9 feeder cells to maintain cell viability during the differentiation process. All scale bars indicate 50 µm. (A, B) Black arrows and arrowheads show enucleated and enucleating cells, respectively. Blue arrows indicate cell debris. Among all HiDEP and HUDEP cell lines, HiDEP-1 cells most efficiently produced enucleated cells.

Before the induction of differentiation, both HiDEP-1 and HUDEP-1 cells had round, erythroblast-like morphologies (Figure 9A, B). After the induction of differentiation in HiDEP-1 cells, enucleating cells (arrowheads in Figure 9A) and enucleated cells (black arrows in Figure 9A) were observed from about 7 days after differentiation. Since the cultures contained various types of cell and cell components such as enucleated cells, extruded nuclei after the enucleation process, nucleated cells, dead cells, and cell debris, it was very difficult to accurately calculate the frequency of enucleated cells even by flow cytometrical and morphological analyses. However, at approximately 12 days after the induction of differentiation a considerable number of enucleated cells were present (Figure 9A). The second cell line, HiDEP-2, could also differentiate into more mature cells and produce enucleated RBCs after the induction of differentiation; however, the efficiency of differentiation was lower compared to HiDEP-1 (data not shown).

After the induction of differentiation in HUDEP-1 cells, the morphology of the cells clearly changed and mature and enucleating cells (arrowheads in Figure 9B) and enucleated cells (black arrows in Figure 9B) were observed from about day 6 of differentiation. Although the numbers of enucleated cells were much lower than those induced from HiDEP-1 cells, we did observe reproducible production of enucleated cells. The other HUDEP cell lines, HUDEP-2 and HUDEP-3, could also differentiate into more mature cells and produce enucleated RBCs after the induction of differentiation; the efficiencies of differentiation were almost identical to HUDEP-1 (data not shown).

### Confirmation of Enucleated Cells

**Figure 10:**
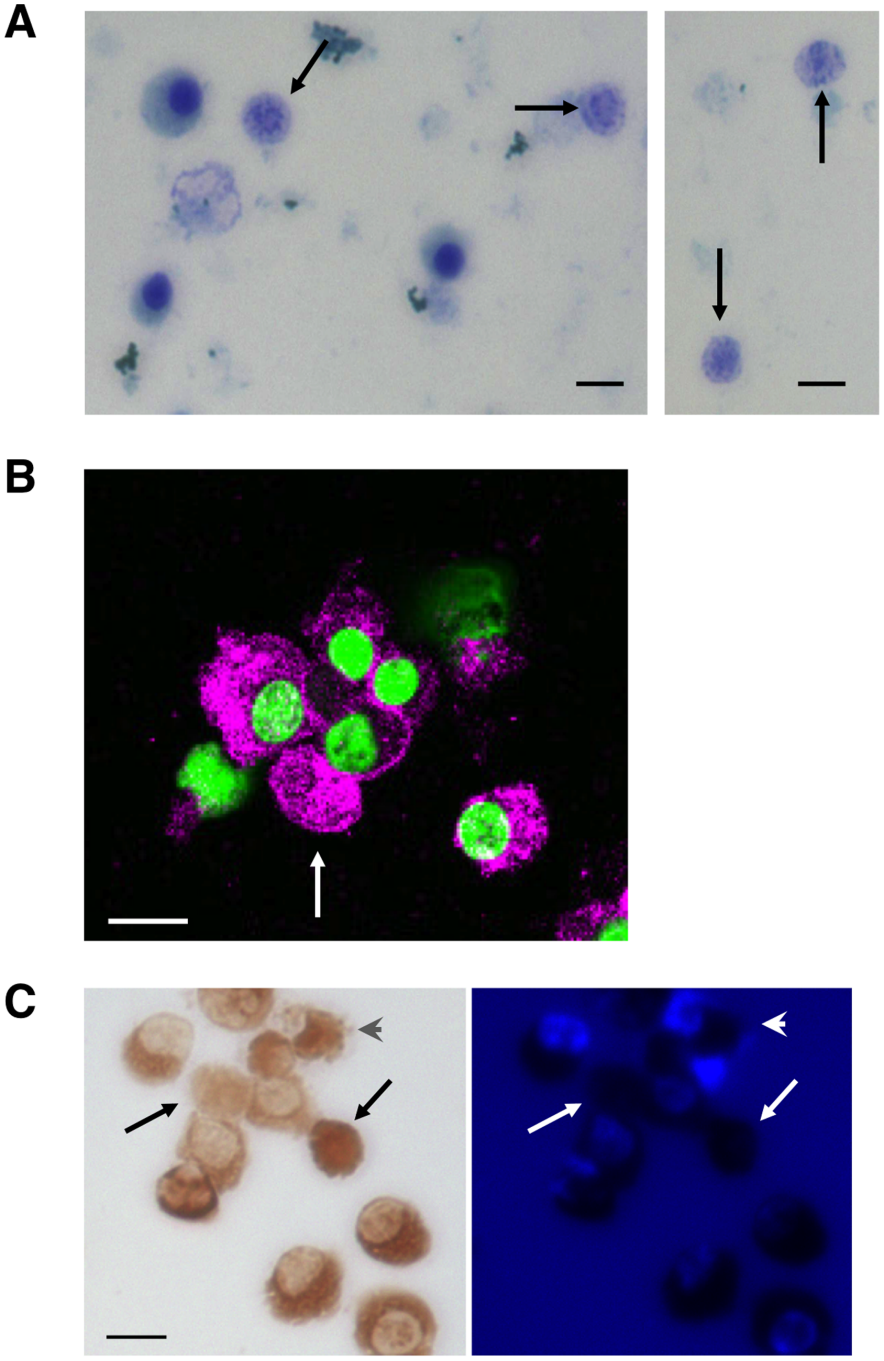
Confirmation of enucleated cells. As a representative, the cells induced from HiDEP-1 cells were subjected to the analyses. (A) Supravital staining. Arrows indicate reticulocytes. (B) Immunohistostaining with glycophorin A antibody (pink) 12 days after the induction of differentiation. Nuclei are stained with SYTO16 (green). An arrow shows an enucleated cell. (C) Benzidine staining 10 days after the induction of differentiation (brown, left panel). After benzidine staining, cell nuclei were labeled with DAPI to distinguish nucleated and enucleated cells (blue stained nuclei in right panel). Arrows or arrowheads show enucleated and enucleating cells, respectively. (A–C) Scale bars indicate 10 µm.

In general, in vitro produced enucleated RBCs are spherical reticulocytes and not fully mature biconcave cells. To confirm that these enucleated cells were reticulocytes, we performed supravital staining, immunostaining and benzidine staining. The supravital staining demonstrated the presence of reticulocytes (arrows in Figure 10A). In addition, we observed GPA-positive enucleated cells (pink cells in Figure 10B) and benzidine-positive enucleated cells (brown cells in Figure 10C).

## Discussion

We previously succeeded in establishing MEDEP cell lines from mouse ES cells and were able to induce enucleated RBC production in these cell lines. However, the protocol developed for mouse cell lines failed to establish immortalized erythroid progenitor cell lines from human ES cells and human iPS cells. This difficulty may be related to the fact that in general it is more difficult to establish immortalized human cell lines than immortalized mouse cell lines. The failure of the original protocol led us to alter our strategy for establishing human erythroid progenitor cell lines.

The principal change to our strategy was to use inducible expression of HPV16-E6/E7 in transfected hematopoietic stem/progenitor cells. Through this approach, we developed a method that enabled establishment of immortalized human erythroid progenitor cell lines that possess the ability to differentiate. In iPS cells, the enforced expression of TAL1 supported the process of obtaining cell lines with hematopoietic potential by increasing the number of induced erythroid cells. To date, we have successfully established immortalized cell lines in all trials with this method. All of the immortalized cell lines we described in this study could differentiate into more mature RBCs including enucleated RBCs. The hemoglobin produced in these cell lines possessed similar oxygen binding properties to the hemoglobin in normal RBCs produced in vivo. To our knowledge, this is the first report describing the establishment of immortalized human erythroid progenitor cell lines able to produce enucleated RBCs ex vivo. Although erythroid progenitor cell lines have previously been established using HPV16-E6/E7, these cell lines did not produce enucleated RBCs [17]. One possible explanation for this failure is the continuous expression of HPV16-E6/E7 in these cells. Since efficient differentiation of cells generally requires cell cycle arrest, the continuous expression of HPV16-E6/E7 might have inhibited terminal differentiation of the cells.

The cell lines obtained in this study displayed different efficiencies for producing enucleated RBCs in vitro as is illustrated by comparison of HiDEP and HUDEP cells. We previously found that the efficiency of enucleated RBC production by MEDEP cells improved in vivo after transplantation [8]. Therefore, it is highly likely that HiDEP and HUDEP cells would also be able to produce enucleated RBCs more efficiently in vivo than in vitro.

Although we suspect that the cell lines established here might be able to produce enucleated RBCs more abundantly in vivo, the main goal of our work is to develop a means of producing abundant enucleated RBCs ex vivo. There are two reasons for this emphasis. First, enucleated RBCs pose no risk of tumorigenicity and, therefore, can be transfused without hazard into the recipient. This is not the case for transplantation of nucleated cells derived from immortalized cells such as ES cells and iPS cells. Enucleated RBCs can be selected by size, e.g., by filtration, and contaminating nucleated cells can be eliminated by irradiation without affecting the structure and function of RBCs. Currently, such irradiation is routinely used in the clinic before transfusion of RBCs in order to eliminate any lymphocytes. Second, transplantation of progenitor cells requires compatibility for major histocompatibility antigens [8]. This is not the case for enucleated RBCs, which mainly require the compatibility of ABO and RhD blood phenotypes. One of the HUDEP cell lines developed here, HUDEP-3, possesses an O/RhD(+) phenotype (Figure S4, S5 and Table S2); thus, RBCs produced ex vivo from this cell line could potentially be transfused into the vast majority of the Japanese population, since approximately 99% of Japanese possess an RhD(+) blood phenotype. Needless to say, cell lines that produce O/RhD(−) RBCs will have more widespread utility as these RBCs can be transfused into the vast majority of people around the world.

Our next aim is to establish cell lines that have a high yield (>90%) of enucleated RBCs after induction of differentiation. The underlying basis for the different characteristics of the various cell lines, such as the efficiency of enucleated RBC production, remains uncertain. Possibly, they may have been immortalized at different stages of erythroid differentiation; the mechanisms of immortalization at different stages of differentiation remain to be studied. Until these mechanisms are elucidated, our experience with the generation of MEDEP, HiDEP and HUDEP cell lines suggests that establishment of useful cell lines will necessitate numerous trials. At present, we do not think that hematopoietic cells derived from iPS cells are a better source than cord blood cells for establishing cell lines. Indeed, HiDEP-1 cells demonstrated a high efficiency of production of enucleated RBCs, while HUDEP-2 cells could produce adult type hemoglobin consisting of α and β globin. We certainly believe it will be feasible to establish HiDEP and/or HUDEP cell lines able to produce adult type hemoglobin and also to produce enucleated RBCs with a high efficiency. With respect to the production of adult type hemoglobin, the results shown in Figure 7 suggested that enforced expression of BCL11A in the source cells may support the establishment of cell lines that produce adult type hemoglobin. In a similar manner to the exogenous expression of TAL1 and HPV16-E6/E7, the introduction of BCL11A will have no consequence for clinical applications if the ultimately established cell lines can produce abundant enucleated RBCs.

Of note, the enucleated cells produced in vitro from HiDEP and HUDEP cells were spherical (Figure 9) and were reticulocytes (Figure 10A). We previously reported that the enucleated RBCs produced from hematopoietic stem/progenitor cells present in cord blood in vitro were also spherical reticulocytes [4]. Generally, the majority of in vitro produced RBCs seem to be reticulocytes; however, it has recently been reported that reticulocytes produced in vitro can complete maturation in vivo, i.e., following transplantation, into biconcave cells [27]. Therefore, it is likely that the reticulocytes produced in vitro from HiDEP and HUDEP cells could also complete maturation in vivo after transfusion.

The method developed to establish the HiDEP cell line involved use of the mouse derived-OP9 cell line as feeder cells during the early stages of establishment and also use of fetal bovine serum (FBS). These xenogeneic factors need to be eliminated before the application of cells in the clinic. There are two reasons for believing this elimination is possible. First, the xenogeneic factors derived from OP9 cells will disappear after long term culture in the absence of OP9 cells. The HiDEP cells ceased to have a requirement for OP9 cells after two or three months in culture and they could be maintained in the presence of humoral factors alone. Notably, HUDEP cell lines could be established in feeder cell-free conditions, i.e., without use of OP9 cells. Second, as we have previously demonstrated, FBS can be replaced with human serum without compromising the production of abundant enucleated RBCs from hematopoietic stem/progenitor cells in cord blood [4]. In addition, all HiDEP and HUDEP cells could be maintained in serum-free conditions using a commercially available medium (see Materials and methods). Thus, with respect to HUDEP cell lines they can be established in a feeder cell-free and serum-free conditions throughout all procedures.

Changes to the characteristics of immortalized cell lines after long term culture can sometimes be an obstacle to their use. However, it is also a fact that many immortalized cell lines, such as human cancer cell lines, stably maintain their characteristic properties even after long term culture. Of note, MEDEP-BRC5, one of the MEDEP cell lines we previously reported [8], has maintained the ability for high rates of production of enucleated RBCs in vitro even after continuous culture for nearly 2 years; in this cell line, more than 50% of the cells are enucleated RBCs after induction of differentiation in vitro [28].

We are now confident that the method described in this study can reproducibly and robustly establish immortalized human erythroid progenitor cell lines, such as the HiDEP and HUDEP cell lines, able to produce enucleated RBCs ex vivo. Establishment of HiDEP cell lines from iPS cells derived from people possessing very rare blood phenotypes, such as Rh-null, may open a way to produce such rare types of RBCs ex vivo.

## Supporting Information

Figure S1 **Map of the CSII-EF-RfA lentiviral vector plasmid.**
(TIF)

Figure S2 **Map of the CSIV-TRE-RfA-UbC-KT lentiviral vector plasmid.**
(TIF)

Figure S3 **Summary of flow cytometry analyses.** Expression of the indicated markers was analyzed. c-KIT, the receptor of SCF. GPA, glycophorin A. (A) Results from HiDEP cells. (B) Results from HUDEP cells.
(TIF)

Figure S4 **Analysis of blood types in HUDEP and HiDEP cells.** (A) The results with respect to the ABO gene. (B) The results with respect to the RhD gene. (A, B) A detailed description of the typing of the cells can be found in Lu et al., Blood 112; 4475–4484 (2008).
(TIF)

Figure S5 **Characterization of phenotype with respect to RhD antigen.** Expression of RhD antigen was clearly detected in HiDEP-1-derived cells. In contrast, HUDEP-3-derived cells did not show abundant expression of RhD antigens. These results indicated that RhD antigens might be induced depending on the stage of maturation of the cells since HiDEP-1 produced mature red blood cells at a higher rate (see manuscript).
(TIF)

Table S1 **Factor dependency of iPS and cord blood-derived erythroid progenitor cell lines.**
(DOC)

Table S2 **Blood phenotypes of the established erythroid progenitor cell lines.**
(DOC)
