# Supplementary figures and images for: Establishment of Immortalized Human Erythroid Progenitor Cell Lines Able to Produce Enucleated Red Blood Cells
Source: PLoS One. 2013 Mar 22;8(3):e59890. doi: 10.1371/journal.pone.0059890 (PMC3606290; doi:10.1371/journal.pone.0059890)

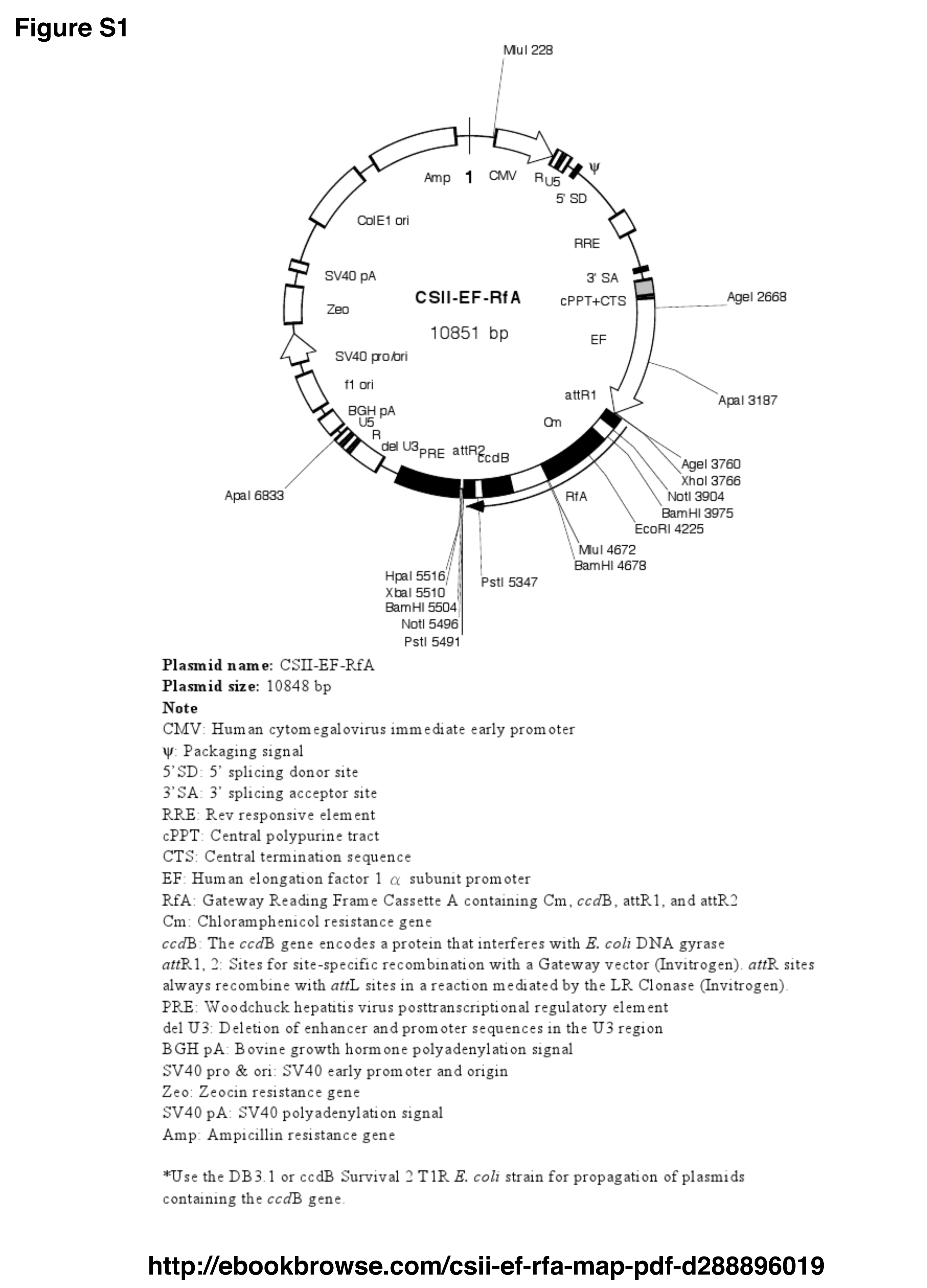

Supplement: Figure S1 — Map of the CSII-EF-RfA lentiviral vector plasmid. (TIF) [file pone.0059890.s001.tif]

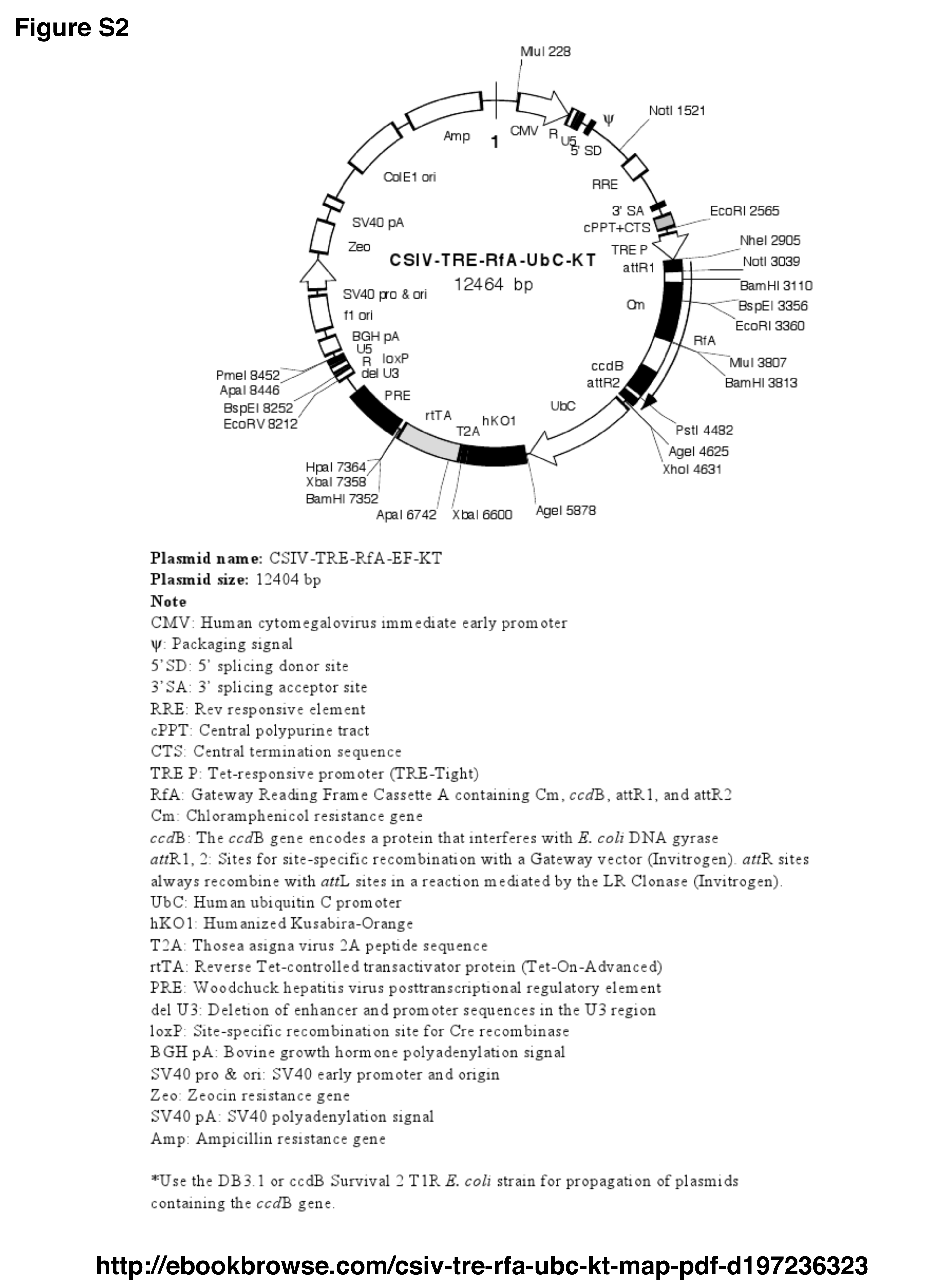

Supplement: Figure S2 — Map of the CSIV-TRE-RfA-UbC-KT lentiviral vector plasmid. (TIF) [file pone.0059890.s002.tif]

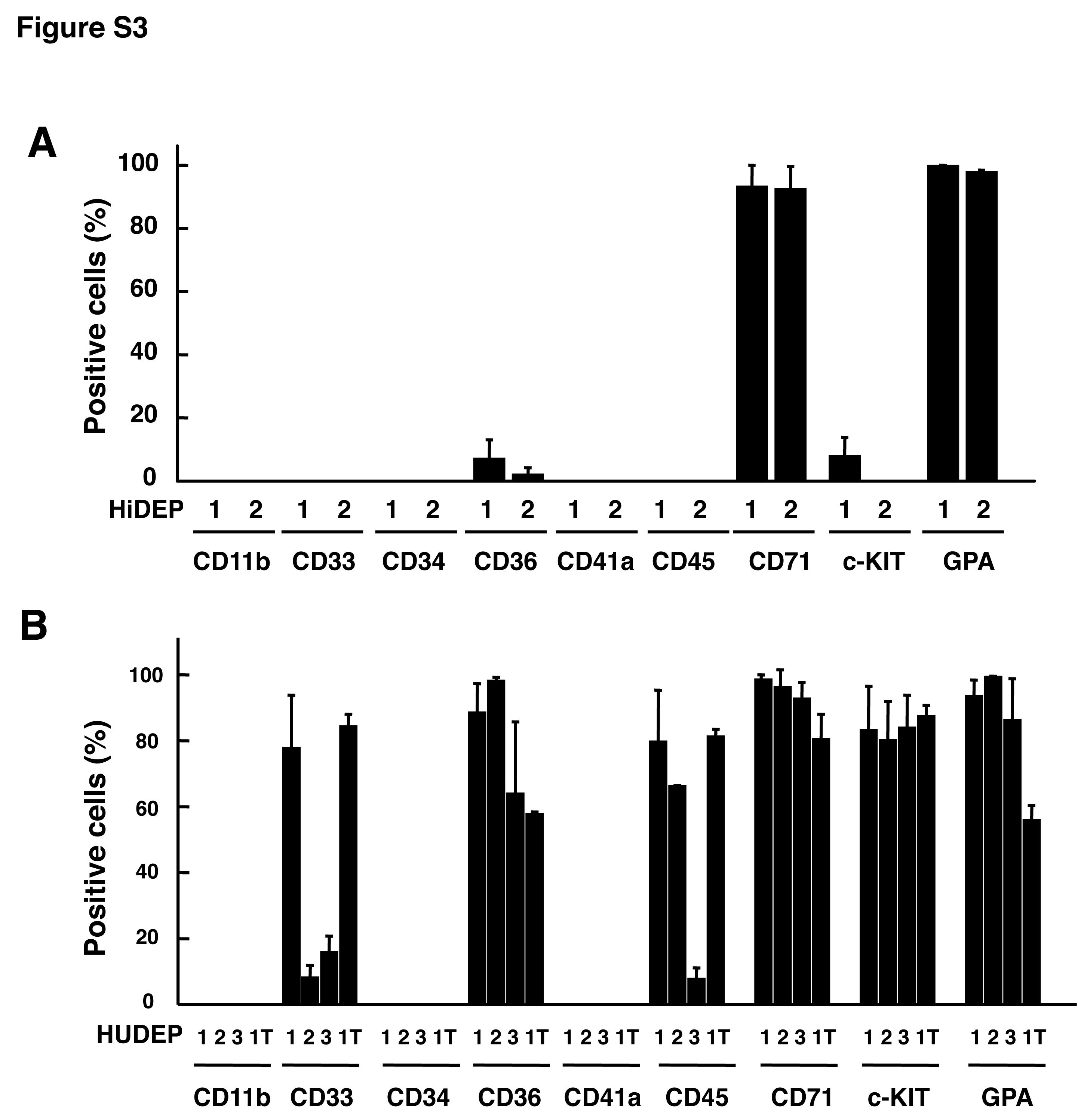

Supplement: Figure S3 — Summary of flow cytometry analyses. Expression of the indicated markers was analyzed. c-KIT, the receptor of SCF. GPA, glycophorin A. (A) Results from HiDEP cells. (B) Results from HUDEP cells. (TIF) [file pone.0059890.s003.tif]

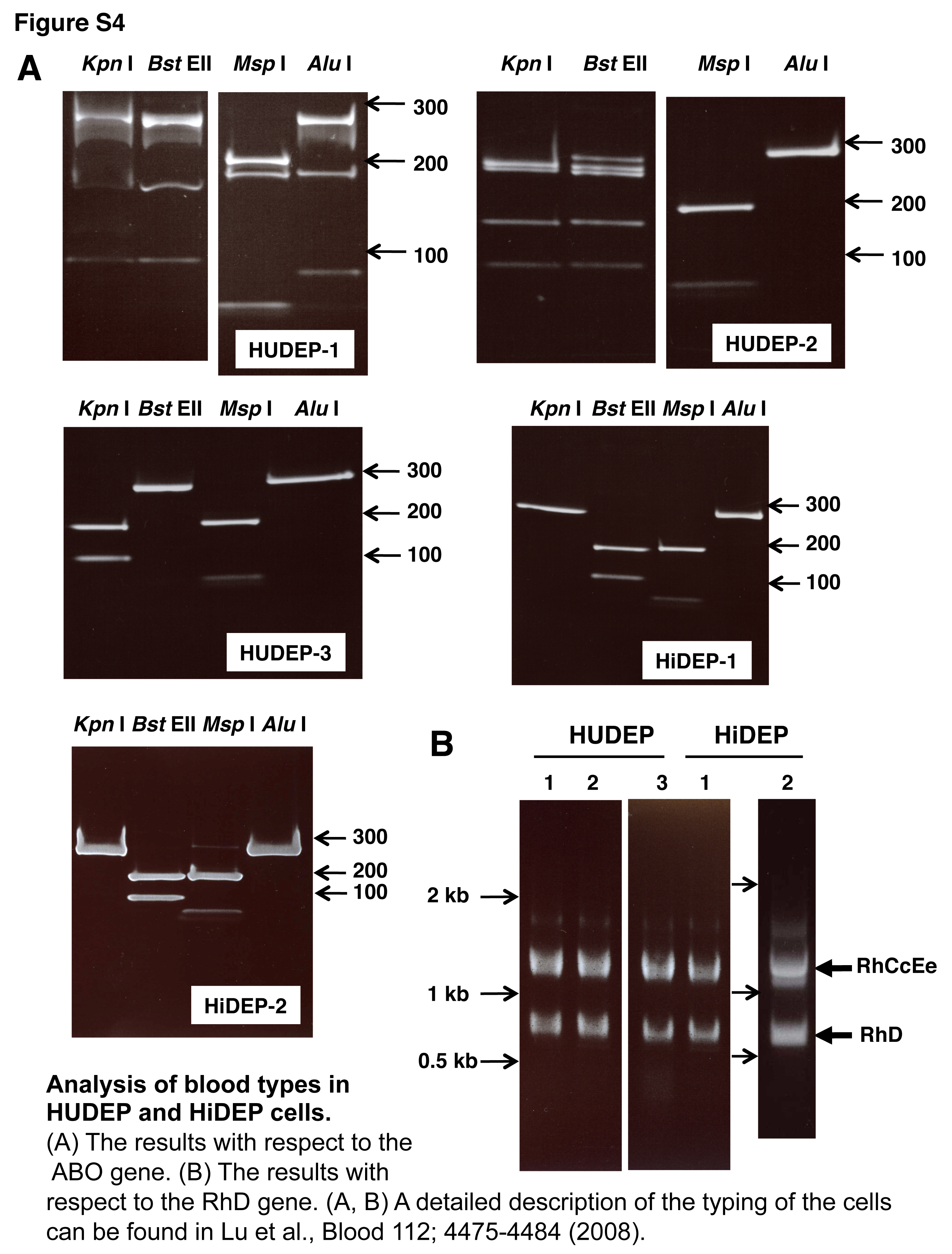

Supplement: Figure S4 — Analysis of blood types in HUDEP and HiDEP cells. (A) The results with respect to the ABO gene. (B) The results with respect to the RhD gene. (A, B) A detailed description of the typing of the cells can be found in Lu et al., Blood 112; 4475–4484 (2008). (TIF) [file pone.0059890.s004.tif]

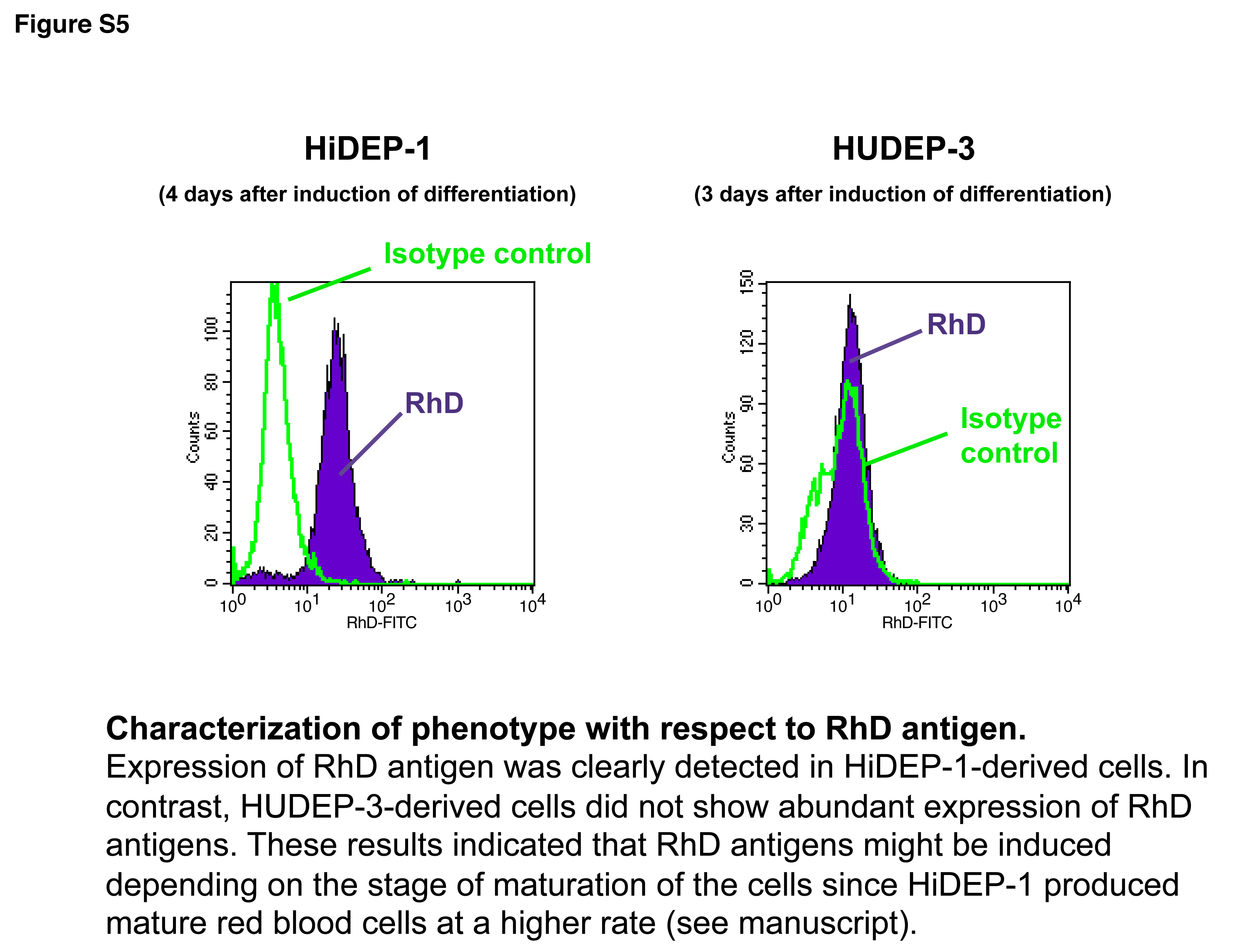

Supplement: Figure S5 — Characterization of phenotype with respect to RhD antigen. Expression of RhD antigen was clearly detected in HiDEP-1-derived cells. In contrast, HUDEP-3-derived cells did not show abundant expression of RhD antigens. These results indicated that RhD antigens might be induced depending on the stage of maturation of the cells since HiDEP-1 produced mature red blood cells at a higher rate (see manuscript). (TIF) [file pone.0059890.s005.tif]
