# Supplementary material for: Establishment of Immortalized Human Erythroid Progenitor Cell Lines Able to Produce Enucleated Red Blood Cells
Source: PLoS One. 2013 Mar 22;8(3):e59890. doi: 10.1371/journal.pone.0059890 (PMC3606290; doi:10.1371/journal.pone.0059890)
Supplement: Table S1 — Factor dependency of iPS and cord blood-derived erythroid progenitor cell lines. (DOC) [file pone.0059890.s006.doc]

Table S1. Factor dependency of iPS and cord blood-derived erythroid progenitor cell lines.

| Cell line | Essential factors for proliferation |
| --- | --- |
| HiDEP-1 | EPO, DOX |
| HiDEP-2 | EPO, DOX |
| HUDEP-1 | SCF, DOX |
| HUDEP-2 | SCF, EPO, DOX |
| HUDEP-3 | SCF, DOX |
