# Supplementary material for: Establishment of Immortalized Human Erythroid Progenitor Cell Lines Able to Produce Enucleated Red Blood Cells
Source: PLoS One. 2013 Mar 22;8(3):e59890. doi: 10.1371/journal.pone.0059890 (PMC3606290; doi:10.1371/journal.pone.0059890)
Supplement: Table S2 — Blood phenotypes of the established erythroid progenitor cell lines. (DOC) [file pone.0059890.s007.doc]

**Table S2.** Blood phenotypes of the established erythroid progenitor cell lines.

| Cell line | ABO genotype | RhD genotype | Blood phenotype |
| --- | --- | --- | --- |
| HiDEP-1 | A/A | + | A/RhD(+) |
| HiDEP-2 | A/A | + | A/RhD(+) |
| HUDEP-1 | B/O | + | B/RhD(+) |
| HUDEP-2 | A/O | + | A/RhD(+) |
| HUDEP-3 | O/O | + | O/RhD(+) |
